# [^18^F]-Fluorinated Carboplatin and [^111^In]-Liposome for Image-Guided Drug Delivery

**DOI:** 10.3390/ijms18051079

**Published:** 2017-05-18

**Authors:** Narottam Lamichhane, Gajanan K. Dewkar, Gobalakrishnan Sundaresan, Rebecca N. Mahon, Jamal Zweit

**Affiliations:** Center for Molecular Imaging, Department of Radiology, Virginia Commonwealth University, 1101 E. Marshall Street, Richmond, VA 23298-0031, USA; lamichhanen@vcu.edu (N.L.); gdewkar@zevacor.com (G.K.D.); sundaresan.gobalakrishnan@vcuhealth.org (G.S.); mahonrn@mymail.vcu.edu (R.N.M.)

**Keywords:** radiolabeled liposomes, radiolabeled platinum, drug delivery, dual modality imaging, PET, SPECT

## Abstract

Radiolabeled liposomes have been employed as diagnostic tools to monitor in vivo distribution of liposomes in real-time, which helps in optimizing the therapeutic efficacy of the liposomal drug delivery. This work utilizes the platform of [^111^In]-Liposome as a drug delivery vehicle, encapsulating a novel ^18^F-labeled carboplatin drug derivative ([^18^F]-FCP) as a dual-molecular imaging tool as both a radiolabeled drug and radiolabeled carrier. The approach has the potential for clinical translation in individual patients using a dual modal approach of clinically-relevant radionuclides of ^18^F positron emission tomography (PET) and ^111^In single photon emission computed tomography (SPECT). [^111^In]-Liposome was synthesized and evaluated in vivo by biodistribution and SPECT imaging. The [^18^F]-FCP encapsulated [^111^In]-Liposome nano-construct was investigated, in vivo, using an optimized dual-tracer PET and SPECT imaging in a nude mouse. The biodistribution data and SPECT imaging showed spleen and liver uptake of [^111^In]-Liposome and the subsequent clearance of activity with time. Dual-modality imaging of [^18^F]-FCP encapsulated [^111^In]-Liposome showed significant uptake in liver and spleen in both PET and SPECT images. Qualitative analysis of SPECT images and quantitative analysis of PET images showed the same pattern of activity during the imaging period and demonstrated the feasibility of dual-tracer imaging of a single dual-labeled nano-construct.

## 1. Introduction

The unique in vivo information provided by molecular imaging has the capacity to improve the overall efficacy of cancer treatment. There is growing demand by physicians, patients, and the community as a whole for personalized healthcare, and molecular imaging is well-positioned to play a major role in the era of precision medicine. Molecular imaging will become more and more multi-modal in nature, as different anatomical and molecular imaging techniques merge to complement each other. Already ^18^F-Fluorodeoxyglucose positron emission tomography ([^18^F]-FDG-PET) is integrated with cross-sectional imaging, in the form of positron emission tomography/computed tomography (PET/CT), which is commonly used in the management of cancer patients. It is inevitable that other molecular imaging technologies, such as molecular resonance imaging/molecular resonance spectroscopy (MRI/MRS), PET, SPECT, and photoacoustic imaging, will be combined to better assess multiple biological factors contributing to disease progression and the working of therapies. With the advancement of targeted multi-modality molecular imaging, it is likely that molecular imaging will provide a personalized molecular fingerprint of individual tumors, as the basis for “tailor made” novel individualized treatment strategies. Furthermore, molecular imaging will rationally facilitate not only the development of new drugs, but also revisit how current drug therapies can be better directed to the individual patient needs in terms of determining the responders and the cost-benefit for each individual patient. Image-guided anti-cancer drug delivery has been under intense recent investigations aimed at better delivery, controlled release, and enhanced retention in the tumor [1,2,3,4,5,6,7]. Radiolabeled pegylated liposomes, encapsulating chemotherapeutic agents, such as doxorubicin, have been investigated in preclinical and clinical settings to monitor the delivery and tissue distribution following systemic administration of drug-encapsulated radiolabeled liposomes [8,9,10,11].

Although this approach enabled imaging of delivery vehicles, in terms of blood circulation and biodistribution kinetics of radiolabeled vehicles, it does not report on the imaging of drug release, uptake, and retention in tumors. Direct in vivo imaging of encapsulated agents requires the incorporation of radiolabeled drugs that can be distinctively imaged from the radiolabeled vehicle. This approach, for example, can be accomplished by radiolabeling a carrier, like liposomes, with a SPECT radionuclide, such as ^111^In or ^99m^Tc, encapsulating a PET radiolabeled drug with ^18^F, for example, or another PET radionuclide. In this way, the dual radiotracer approach will be more informative in that the right amount of drug reaching and retaining in the tumor target can be directly and quantitatively determined from the imaging data. It will also be capable of directly relating the in vivo biodistribution kinetics of both the radiolabeled drug and the radiolabeled liposomal carrier. Liposomes, as drug carriers, have been radiolabeled with various SPECT radionuclide chelates and their biodistribution and pharmacokinetics were studied by imaging [12,13,14,15,16,17,18,19]. Recent work has also exploited longer-lived PET radionuclides, such as ^64^Cu, ^89^Zr, and ^52^Mn, which were used to radiolabel liposomes [20,21,22,23].

**Figure 1:**
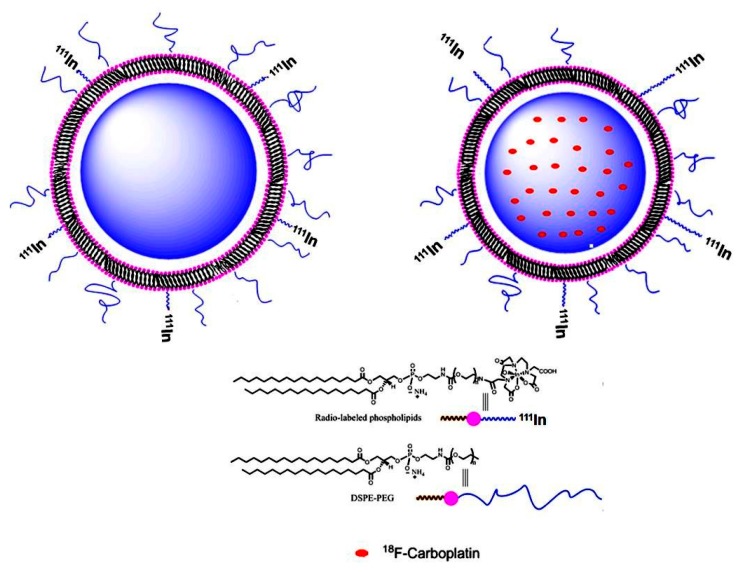
Schematics showing [^111^In]-Liposome and its components along with encapsulated [^18^F]-Carboplatin; DSPE-PEG: 1,2-distearoyl-*sn*-glycero-3-phosphoethanol-amine-*N*-[methoxy(polyethyleneglycol)] ammonium salt.

In this work, a radiolabeled fluorinated carboplatin derivative ([^18^F]-FCP) was encapsulated inside an [^111^In]-Liposome to enable distinct molecular imaging by PET and SPECT, respectively. A schematic of this approach is depicted in Figure 1. This strategy could be more informative in imaging drug delivery, in that the in vivo biodistribution profile of both the drug and the vehicle can be distinctively imaged in the same subject. To the best of our knowledge, this is the first study that incorporates a radiolabeled platinum drug derivative encapsulated in radiolabeled liposomes.

## 2. Results

### 2.1. Analysis

The surface labeling efficiency of liposome with ^111^In was greater than 90% after 30 min at 40 °C for both the [^111^In]-Liposome and [^18^F]-FCP encapsulated [^111^In]-Liposome. The mean diameter of [^111^In]-Liposome was 168 ± 17 nm, and the ζ potential was −2.3 ± 0.19 mV. The average diameter of [^18^F]-FCP encapsulated [^111^In]-Liposome was 221 ± 20 nm with a ζ potential of −2.11 ± 0.98 mV. The encapsulation efficiency of [^18^F]-FCP in the liposomal formulation was 38 ± 2% (*n* = 3).

### 2.2. Biodistribution of [^111^In]-Liposome

Biodistribution data of [^111^In]-Liposome are summarized in Table 1. In the first hour, prominent uptake of [^111^In]-Liposome in major organs, including the blood, liver, spleen, lungs, kidneys, and heart, was observed. The level in the blood was reduced seven-fold by 6 h, and further reduced to nearly 200-fold by 48 h. Blood circulation half-life was calculated to be 6.2 h. Gradual reduction of radioactivity in all major tissues, with most of the radioactivity, is due to the liver and spleen two days after injection. Uptake of this size of liposomal formulation is typical of reticuloendothelial system (RES) accumulation with gradual clearance.

### 2.3. SPECT/CT Imaging of [^111^In]-Liposome

**Figure 2:**
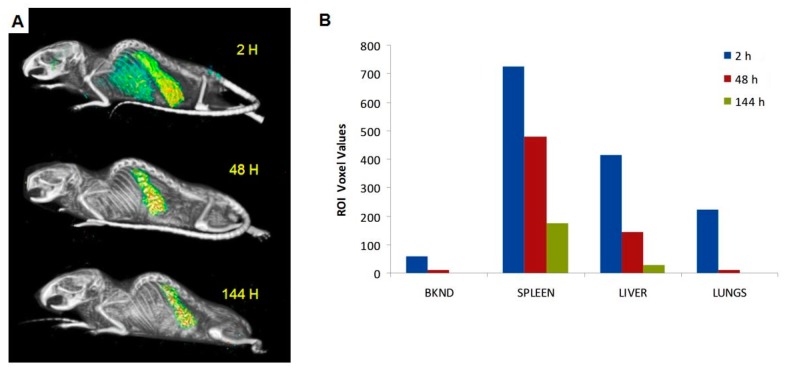
In vivo SPECT images (**A**) of normal nude mouse injected with [^111^In]-Liposome through the tail vein and imaged at 2, 48, and 144 h post-injection. Initial high uptake in the RES tissues was significantly reduced at later time points. Relative radioactive signal intensity of [^111^In]-Liposome in the spleen, liver, and lungs, compared to a background region on the image, is shown. All regions were determined by regions of interest (ROI) analysis of SPECT images (**B**).

SPECT/CT images (Figure 2) of [^111^In]-Liposome mirrors that of the ex vivo biodistribution data. The overall picture of the radioactive signal in the 2 h image reflects a similarity to the ex vivo biodistribution at 1 h post injection. Subsequent clearance of radioactivity from thoracic region was observed over time with the remaining radioactivity after six days was predominately due to the spleen and liver, consistent with ex vivo data. At six days post-injection, the radioactivity in the liver and spleen was 16 and four times lower than that at 2 h, respectively.

### 2.4. PET/SPECT/CT Imaging

**Figure 3:**
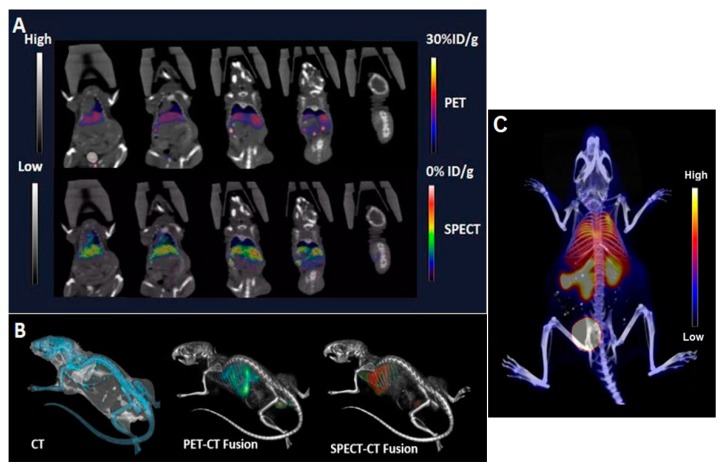
In vivo computed tomograpghy (CT), positron emission tomography (PET)/CT, and SPECT/CT images of a nude mouse injected with 14 MBq of [^18^F]-FCP encapsulated [^111^In]-Liposome through tail vein injection 1 h post-administration. Both PET/CT and SPECT/CT images show the uptake of [^18^F]-FCP encapsulated in [^111^In]-Liposome in the liver and spleen. Both images correspond to each other in the uptake profile, demonstrating the feasibility of dual-tracer imaging from a single nano-construct. (**A**) Coronal images, (**B**) volume rendered images, and (**C**) maximum intensity projection (MIP) image 1 h post-injection.

PET/CT and SPECT/CT imaging (Figure 3) was carried out in the same animal injected with radiolabeled [^18^F]-FCP encapsulated [^111^In]-Liposome. Images acquired 1 h post-injection reflect radioactivity mainly in the RES organs. No cross-detection of the SPECT signal from ^111^In was visually noted in the PET images, indicating efficient energy window thresholding. One hour post-injection, SPECT images were acquired using 171 keV γ’s from ^111^In and were automatically fused to CT. Like the PET/CT images, and consistent with ex vivo biodistribution data, the SPECT/CT images demonstrated a very similar pattern of radioactivity accumulation in the RES (Figure 3A). This shows that 1 h post-injection of the [^18^F]-FCP encapsulated [^111^In]-Liposome, the image profile is very similar to that of radiolabeled liposomes indicating that the ^18^F radioactivity (and, hence, [^18^F]-FCP) is intact within the liposomal formulation.

**Figure 4:**
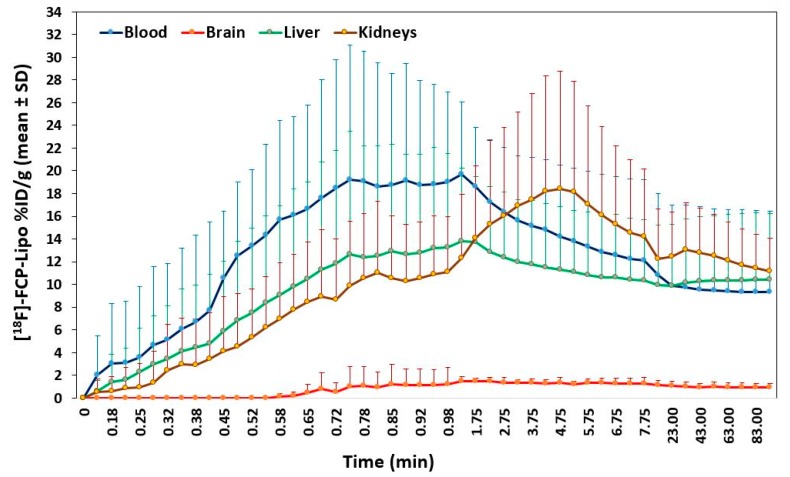
Time-activity curve data (*n* = 3) generated from the dynamic PET image dataset of [^18^F]-FCP encapsulated liposomes shows the time-dependent kinetics and tissue clearance in mice.

Tracer metabolic assessment using the dynamic time activity measurement of [^18^F]-FCP encapsulated liposome over regions of the blood, liver, kidney, and brain, as low signal regions, are shown in Figure 4. High uptake persisted early in the blood and reached a maximum between 1–2 min and stabilized after 20 min. An increased activity trend was observed more over the liver than the kidney early after administration, indicating greater RES uptake. Comparative uptake of [^18^F]-FCP encapsulated liposome in the kidney was much less than that of naked [^18^F]-FCP (data not shown).

### 2.5. Optimization of Dual Radionuclide Imaging of ^111^In and ^18^F

**Figure 5:**
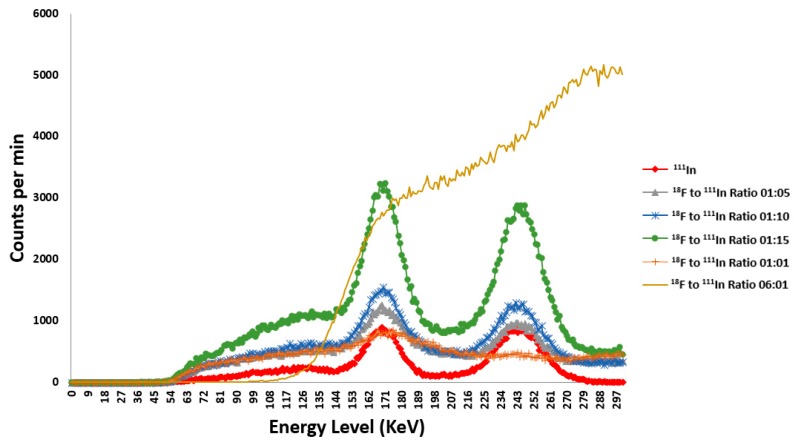
^18^F and ^111^In spectrum comparison at different ratios.

The subtraction window with the best recovery of the ^111^In spectrum was found to be 5% for the three different ratios. The data acquired from the ratios of the ^18^F to ^111^In yielded spectra that resembled the combined ^18^F and ^111^In for 1:5, 1:10 and 1:15 ratios with the 1:1 ratio resembling the spectrum of ^18^F alone as seen in Figure 5. The 1:1 and 6:1 (^18^F:^111^In) curve shows an indistinguishable ^111^In curve. The 6:1 ratio appears to saturate the detector. This could be because the activity of the PET isotope is too high, and it is increasing the dead time in the SPECT detector or even flooding the camera [24]. In our experiment, no bleed through of 511 keV downscattered photons was observed in SPECT images. This finding can be attributed to the radioactivity mixture ratio of 9:1 of ^111^In and ^18^F in our liposomal drug construct which was similar to reported finding by Bartoli et al. [25].

## 3. Discussion

Imaging with radiolabeled liposomes has been applied in a broad range of applications. ^111^In with a half-life of 2.8 days is well suited to monitor the fate of liposomes using in vivo SPECT imaging for a longer duration. In vivo behavior of liposomes depends on the components of the liposomes. To understand the detailed characteristics on the in vivo behavior of the [^111^In]-Liposome, we also studied the components of the liposomes that were used to synthesize the liposomes for this study (data not shown). Detailed in vivo biodistribution of [^111^In]-Liposome varied directly with the behavior of the components of liposome. Biodistribution of ^111^InCl_3_ showed major accumulation of the activity in the liver and spleen in the first hour of administration. Subsequent clearance of activity from the thoracic region and increased accumulation of activity in the kidneys was observed in later time points. [^111^In]-Diethylenetriaminepentaacetic acid ([^111^In]-DTPA) cleared very rapidly from the circulation with most activity observed in kidneys within an hour of administration, suggesting renal clearance of the radiotracer, and agreeing with the data published by Harrington et al. [13].

In this work, we demonstrated a proof of concept of two modalities, PET and SPECT imaging of a radiolabeled drug encapsulated in a radiolabeled liposome. [^18^F]-FCP was efficiently encapsulated in [^111^In]-Liposome and the formulation was imaged in both PET/CT and SPECT/CT modes. Energy discrimination capability was used to avoid cross-detection of ^111^In SPECT activity in the ^18^F PET image. The biodistribution profile of the [^111^In]-Liposome showed major uptake in the spleen and liver. In vivo SPECT imaging with [^111^In]-Liposome mirrored the ex vivo biodistribution data with major accumulation in spleen and liver. Images during later time points (48 and 144 h) showed the gradual clearance of activity from the liver and spleen. In addition, we have demonstrated the feasibility of dual-tracer sequential imaging with PET and SPECT using [^18^F]-FCP encapsulated [^111^In]-Liposome. [^18^F]-FCP encapsulated [^111^In]-Liposome showed major uptake in RES in both PET and SPECT images. Qualitative analysis of SPECT image enabled by ^111^In corresponded with PET image enabled by ^18^F demonstrating the feasibility of dual-tracer imaging from the single nano-construct. With [^111^In]–Liposome, we observed the uptake of liposome in RES, which was also propagated in PET and SPECT images acquired from [^18^F]-FCP encapsulated [^111^In]-Liposome in an hour after administration. Multimodal imaging has been very useful for researchers for a range of in vivo studies. Single-modality imaging, such as SPECT and PET, has been intensively used to gather in vivo functional data. Disease states, and the unique pathways they express, may not be easily visible for a single modality. Therefore, dual-modality imaging further strengthens the capacity to gather robust data and can image different pathways simultaneously/sequentially, which is otherwise not possible with a single modality.

Liposomes have a wide range of properties that enable their surface functionalization to attach different radioisotopes that can be used for drug delivery to imaging tumors. Despite the established potentials of liposomes as drug delivery vehicles, liposomal drugs are not effective to all tumors. Development of biomarkers to identify responder patients to liposomal drugs is crucial [10]. The effectiveness of liposomal agents depends on the in vivo accumulation levels of these agents in tumor. The combination of PET and SPECT isotopes with shorter and longer half-life in a single hybrid platform enables in vivo tracking of these two isotopes using different modalities for various time points. This helps in characterizing a disease correlating distinct information from a dual modality in interrogating disease to further optimize the treatment in an individual basis. Various researchers have presented dual-modality imaging with a combination of reporters and tracers. Chapman et al. [26] demonstrated the dual-tracer imaging feasibility of SPECT and PET probes in living mice using a sequential protocol. As part of the dosing protocol in the study Chapman et al. injected SPECT tracer at first and acquired the SPECT images and injected the PET tracer after the SPECT scan was performed. Matsunari et al. [27] studied the detection of viable myocardium using dual isotope simultaneous acquisition SPECT using [^18^F]-FDG and [^99m^Tc]-sestamibi. However, there are very few studies that combine both PET and SPECT radiotracers in a single nanoparticle.

Dual PET/SPECT imaging can greatly enhance the ability to trace encapsulated drugs as they moved throughout the body. By tagging the encapsulating substance and the drug separately, SPECT/PET imaging can be used to monitor the effectiveness of encapsulated drug uptake in the body, as well as actual drug delivery. The development of novel [^18^F]-FCP encapsulated [^111^In]-Liposome facilitates the delivery of platinum drugs preferentially to tumor sites by altering the toxicity profile of the entrapped agent. This theranostic approach of delivering radiolabeled platinum drugs enables non-invasive, real-time visualization of drug fate in vivo. The feasibility of monitoring the drug enables the study of its pharmacokinetics and accumulation in real-time. The real-time monitoring of both the liposomal vehicle using SPECT and platinum drug using PET provides added information on the behavior of both the vehicle and drug. Heterogeneity of various diseases within, and between the patients may not always be feasible to study with known radiolabeled drugs. [^18^F]-FCP alone or inside liposomes enables an all-in-one concept where the combination of theranostic drugs and imageable delivery can be used for both systemic and localized delivery, as well as imaging agents.

## 4. Materials and Methods

### 4.1. Synthesis of [^111^In]-Liposome

All chemicals were used as received without further purification. 1,2-dipalmitoyl-*sn*-glycero-3-phosphocholine (DPPC), 1,2-dipalmitoyl-*sn*-glycero-3-phosphoethanolamine-*N*-diethylenetriaminepentaacetic acid (ammonium salt) (DPPE-DTPA) were purchased from Avanti Polar Lipids (Alabaster, AL, USA). 1,2-distearoyl-*sn*-glycero-3-phosphoethanol-amine-*N*-[methoxy(polyethyleneglycol)-2000] ammonium salt (DSPE-PEG_2000_) was bought from Laysan Bio, Inc. (Arab, AL, USA). Cholesterol (Chol) was purchased from Sigma-Aldrich (Steinheim, Germany).

Pegylated liposome (Figure 1) was synthesized using the method described my Mougin-Degraef et al. [17]. DPPC, Chol, DSPE-PEG_2000_, DPPE-DTPA (60:30:5:5 mole ratio) were mixed in a 10-mL vial and dissolved with chloroform:methanol (CHCl_3_:MeOH, 9:1). The solvent was evaporated under rotary evaporation to form a lipid film followed by high vacuum to remove the residual organic solvent. Dulbecco’s phosphate-buffered saline (PBS; 1 mL) was added and the suspension was sonicated at 50–60 °C for 10 min. At the end of the incubation, the liposome solution was immediately chilled in ice. The resulting vesicles were extruded through a series of polycarbonate membrane at 50–60 °C. The extruded liposomes were purified using gel chromatography on Sephadex G-25 (Sigma-Aldrich, Steinheim, Germany) with PBS as an eluent.

Pegylated liposome was labeled with ^111^In as previously described by Chow et al. [15]. Briefly, 500 µL of preformed liposome was incubated with 20 µL of ^111^InCl_3_ (indium chloride in 0.05 M HCl; 3.7–74 MBq) (Perkin Elmer, Waltham, MA, USA/Triad Isotopes, Richmond, VA, USA) in 20 µL of 3 M sodium acetate buffer (pH 5.2) and then incubated at 40 °C for 30 min. Unincorporated ^111^In was purified using gel chromatography (Sephadex G-25; Sigma-Aldrich, Steinheim, Germany). The labeling efficiency was determined by measuring the activity in dose calibrator (CRC-15R, Capintec; Bioscan, Florham Park, NJ, USA) for the liposome, filter, and supernatant.

### 4.2. Synthesis of [^18^F]-FCP

[^18^F]-FCP was synthesized using an automated synthesis method using an Allinone synthesizer (Trasis SA, Ans, Belgium). The method has been developed in collaboration with Trasis, S.A., Ans, Belgium, and is under review for publication elsewhere. Radio synthesis of [^18^F]-2-(5-fluoro-pentyl)-2-methyl malonic acid ([^18^F]-FPMA) was performed as per our previously-published procedure [28]. “Briefly, radiosynthesis of [^18^F]-FPMA was carried out using the tosylate precursor. [^18^F] received from a cyclotron (GEMedical Systems, Wausheka, WI, USA), and was passed through preconditioned QMA Sep-Pak (Waters Corp., Milford, MA, USA). The trapped [^18^F] was eluted with 0.08 mL of potassium carbonate (0.025 mol) and 0.42 mL of sterile water into 10 mL V vial containing a solution of Kryptofix (12 mg) in dry acetonitrile (0.8 mL). The reaction mixture was dried. Four milligrams of tosylate precursor in acetonitrile was added to the dry mixture and was heated at 110 °C for 10 min. The reaction mixture was cooled and dried. Base hydrolysis was performed with methanolic NaOH solution in 2 mL of (DCM:MeOH; 9:1) for 20 min at 45 °C. After solvent evaporation sodium citrate buffer (pH 3) and 3 mol HCl (1.5:0.5 mL) was added. The reaction mixture was passed through a preconditioned C18 column (Waters Corp, Milford, MA, USA) and eluted with 2 mL ethanol. The ethanol was evaporated at 45 °C under vacuum and helium flow. The purified [^18^F]-FPMA was dissolved in water. Cisplatin aqua complex was coordinated with [^18^F]-FPMA at 75 °C for 30 min to produce a reaction mixture of [^18^F]-FCP and [^18^F]-FPMA. The reaction mixture was purified by using QMA Sep-Pak ion-exchange column (Waters Corp., Milford, MA, USA) to produce [^18^F]-FCP” [29].

### 4.3. [^18^F]-FCP encapsulation in [^111^In]-Liposomes

DPPC, Chol, DSPE-PEG_2000_, DPPE-DTPA (60:30:5:5 mole ratio) were mixed in a 10 mL vial and dissolved with chloroform:methanol (CHCl_3_:MeOH, 9:1). The solvent was evaporated under a rotary evaporator to form a lipid film followed by high vacuum to remove the residual organic solvent. Freshly synthesized [^18^F]-FCP in PBS was added to the lipid film and was sonicated for 10 min at 60 °C. At the end of sonication, the liposome solution was immediately chilled in ice. For the radiolabeling with ^111^In, sodium acetate buffer (3 M, pH 5.2) was added to liposome solution. ^111^InCl_3_ was buffered with sodium acetate buffer (3 M, pH 5.2) for 2 min before incubating with buffered liposome solution at 40 °C for 30 min. The resultant liposome nano-construct was purified using gel chromatography (Sephadex G-25; Sigma-Aldrich, Steinheim, Germany). The purified liposome nano-construct was further used for characterization and in vivo studies. Zeta Sizer Nano Series ZEN3600 (Malvern, UK) was used to measure the hydrodynamic size and ζ potential of liposomes in PBS (pH ~7.2). The radioactive reaction yield was calculated from gamma counting. The labeling and encapsulation efficiency were determined by counting the liposome suspension before and after chromatography on a PD-10 (Sephadex G-25; Sigma-Aldrich, Steinheim, Germany) with a dose calibrator (CRC-15R, Capintec; Bioscan; Florham Park, NJ, USA). The encapsulation efficiency of [^18^F]-FCP was calculated as percentage of radioactivity that was entrapped inside the liposome to the initial radioactivity of [^18^F]-FCP.

### 4.4. Biodistribution of [^111^In]-Liposome

Animal experiments were approved and performed according to the policies and guidelines of the Institutional Animal Care and Use Committee (IACUC) (AD20170) at Virginia Commonwealth University. Adult female nude mice were injected with [^111^In]-Liposome through tail vein. The amount of radiotracer injected was (1.07 ± 0.03 MBq) in 200 µL PBS. At different time points post injection (1 h, 6 h, 48 h), mice were euthanized, and key tissues were harvested. Tissues were counted in a gamma counter and % ID/g of the tissue was determined.

### 4.5. In vivo SPECT/CT Imaging of [^111^In]-Liposome

Micro-SPECT was performed using a multimodal (DPET/SPECT/CT) preclinical imaging system (Siemens, Knoxville, TN, USA). Nude mice were intravenously injected with [^111^In]-Liposomes (8 MBq, 200 μL) and whole body micro-SPECT imaging was carried out in prone position. Imaging was carried out using 171 keV energy gammas 2 h, 48 h and 144 h post injection. Micro-CT was also performed with 75 kV and 500 μA at a resolution of 96 μm. The whole-body scan time was 10 min. The SPECT images were reconstructed using an iterative reconstruction algorithm (ordered-subset expectation maximization or OSEM3D) modified for the five-pinhole geometry with a 20% energy window around the 171 keV photo peak of ^111^In. These images were then registered with CT images based on a transformation matrix previously generated using four ^57^Co land markers. Images were viewed and quantified using Inveon Research Workplace (IRW) software (IAW 1.6, Siemens, Knoxville, TN, USA). ROIs covering the entire organs were drawn and the average counts were measured and the data was correlated to ex vivo biodistribution result from gamma counting.

### 4.6. PET/SPECT/CT of [^18^F]-FCP/[^111^In]-Liposome

Female nude mice (Harlan Laboratories, Indianapolis, IN, USA), aged four to six weeks, were employed for the in vivo imaging.

[^18^F]-FCP encapsulated Liposomes (5.5–6 MBq) was injected intravenously in a normal female nude mouse via the tail vein. Immediately following the injection, a 90-min dynamic scan was performed. PET images were reconstructed using the Fourier Re-binning and Ordered Subsets Expectation Maximization (OSEM) 3D algorithm with dynamic framing. Reconstructed PET images were co-registered with CT images and analyzed using IRW software (IAW 1.6, Siemens, Knoxville, TN, USA).

[^18^F]-FCP encapsulated in [^111^In]-Liposome was administered via tail vein injection. A nude mouse was injected with total activity of 15 MBq of [^18^F]-FCP/[^111^In]-Liposome (9:1; ^111^In:^18^F ratio). One hour post-administration, the mouse was anesthetized by isoflurane (2.0% flow rate) and kept under nose cone set up for imaging. Static summed up PET image data 60 min post injection was acquired for 30 min. At completion of the PET imaging, without moving the specimen, the mouse bed was moved to the SPECT/CT imaging planes. A SPECT scan was performed using dual-head camera mounted with 2 multi-pinhole collimators (five 1.0-mm pinholes in each collimator, 51-mm transaxial FOV, 40-mm radius of rotation, and a maximum resolution of 1.5 mm. Images were acquired over 360° in a total of 40 projections, resulting in a total imaging time of 60 min. Micro-CT was also performed with 75 kV and 500 μA at a resolution of 96 μm. The whole-body scan time was 10 min. Administration of a cocktail of [^18^F] and [^111^In] radioactivity allowed for conserving the same animal position to fix the spatial position for both SPECT and PET imaging modes.

### 4.7. Optimization of Dual-Radionuclide Imaging of ^111^In and ^18^F

In order to minimize the degradation of the SPECT image due to downscatter from the 511 keV used to produce the PET image, phantom experiments with different radioactivity ratios of ^111^In and ^18^F were carried out on the Inveon PET/CT/SPECT preclinical scanner (Siemens, Knoxville, TN, USA). The ^18^F to ^111^In ratio pairs tested were 6:1, 1:1, 1:5, 1:10, and 1:15. The activities totals were kept below the saturation level for the detectors and around a reasonable injection activity for a mouse study. The primary source degrading the SPECT photopeak comes from the backscatter events from the 511 keV annihilation photons. The subtraction procedure used was based on Bartoli et al. [25], and was applied to the spectrum scan from the simultaneously present ^111^In and ^18^F scan to determine the ability to recover the ^111^In alone spectrum scan. With preclinical scanner used, SPECT, PET, and CT scans are taken sequentially and cannot be performed simultaneously. During all imaging scans, the phantom maintains the same geometry as the bed position is moved from the SPECT/CT scanner to the docked PET scanner within the Inveon scanner system.

## 5. Conclusions

DTPA-derivatized pegylated liposome was labeled with ^111^In. [^111^In]-Liposome was evaluated in vivo with biodistribution and SPECT imaging. [^18^F]-FCP was encapsulated in [^111^In]-Liposome. Dual-tracer feasibility of PET/SPECT imaging with [^18^F]-FCP encapsulated in [^111^In]-Liposome was demonstrated.

## Figures and Tables

**Table 1:** Biodistribution of tail-vein injected [^111^In]-Liposome (1.07 MBq ± 0.03) in nude mice (*n* = 3) per time point.

Organs	1 h	6 h	48 h
Blood	26.72 ± 8.80	3.74 ± 0.49	0.15 ± 0.01
Heart	5.83 ± 0.73	1.11 ± 0.05	0.21 ± 0.00
Lungs	7.63 ± 1.01	1.97 ± 0.20	0.34 ± 0.00
Liver	15.05 ± 1.07	22.86 ± 3.86	7.71 ± 0.44
Spleen	65.92 ± 4.65	73.33 ± 12.13	45.01 ± 2.94
Stomach	0.70 ± 0.22	0.35 ± 0.07	0.20 ± 0.05
Intestine	2.12 ± 0.68	1.24 ± 0.13	0.66 ± 0.06
Kidneys	4.45 ± 0.35	1.62 ± 0.33	0.75 ± 0.05
Skin	1.39 ± 0.21	0.89 ± 0.18	0.62 ± 0.08
Muscle	0.73 ± 0.21	0.39 ± 0.09	0.23 ± 0.03
Skull	3.35 ± 0.48	0.94 ± 0.21	0.28 ± 0.04
Brain	0.60 ± 0.01	0.13 ± 0.01	0.01 ± 0.00
Femur	1.28 ± 0.10	0.99 ± 0.18	0.6 ± 0.10

Data are presented as the percent injected dose per gram (%ID/g) (mean ± standard error of mean (SEM) for *n* = 3) values determined through gamma counting. Tissues were collected, weighed, and the gamma emission was measured in a gamma counter to calculate %ID/g.
